# Exploring agro-morphological and fiber traits diversity in cotton (*G. barbadense* L.)

**DOI:** 10.1186/s12870-024-04912-0

**Published:** 2024-05-15

**Authors:** Ehab A. A. Salama, Mona A. Farid, Youssef A. El-Mahalawy, A. A. A. El-Akheder, Ali A. Aboshosha, Aysam M. Fayed, W. M. B. Yehia, Sobhi F. Lamlom

**Affiliations:** 1 Agricultural Botany Department, Faculty of Agriculture (Saba Basha), Alexandria University, Alexandria, 21531 Egypt; 2 Genetics Department, Faculty of Agriculture, Kafr El-Sheikh University, Kafr El-Sheikh, Egypt; 3 Cotton Breeding Department, Agriculture Research Center, Cotton Research, Cotton Research Institute, Kafr El-Sheikh, Egypt; 4 Molecular Biology Department, Genetic Engineering and Biotechnology Institute, University of Sadat City, Sadat, 32897 Egypt; 5 Plant Production Department, Faculty of Agriculture (Saba Basha), Alexandria University, Alexandria, 21531 Egypt

**Keywords:** Cotton, Yield, Genetic diversity, SSR, DNA fingerprinting, Fiber traits

## Abstract

Cotton (*Gossypium barbadense* L.) is a leading fiber and oilseed crop globally, but genetic diversity among breeding materials is often limited. This study analyzed genetic variability in 14 cotton genotypes from Egypt and other countries, including both cultivated varieties and wild types, using agro-morphological traits and genomic SSR markers. Field experiments were conducted over two seasons to evaluate 12 key traits related to plant growth, yield components, and fiber quality. Molecular diversity analysis utilized 10 SSR primers to generate DNA profiles. The Molecular diversity analysis utilized 10 SSR primers to generate DNA profiles. Data showed wide variation for the morphological traits, with Egyptian genotypes generally exhibiting higher means for vegetative growth and yield parameters. The top-performing genotypes for yield were Giza 96, Giza 94, and Big Black Boll genotypes, while Giza 96, Giza 92, and Giza 70 ranked highest for fiber length, strength, and fineness. In contrast, molecular profiles were highly polymorphic across all genotypes, including 82.5% polymorphic bands out of 212. Polymorphism information content was high for the SSR markers, ranging from 0.76 to 0.86. Genetic similarity coefficients based on the SSR data varied extensively from 0.58 to 0.91, and cluster analysis separated genotypes into two major groups according to geographical origin. The cotton genotypes displayed high diversity in morphology and genetics, indicating sufficient variability in the germplasm. The combined use of physical traits and molecular markers gave a thorough understanding of the genetic diversity and relationships between Egyptian and global cotton varieties. The SSR markers effectively profiled the genotypes and can help select ideal parents for enhancing cotton through hybridization and marker-assisted breeding.

## Introduction

Cotton (*Gossypium* L.) is an important economic and fiber crop grown annually in over eight countries including the USA, India, China, and Egypt. It provides over 95% of the raw natural fibers used in the textile industry and has value as a bioenergy and oilseed crop [1, 2]. Cotton belongs to the *Gossypieae* tribe in the *Malvaceae* family, comprising about 53 species including 46 diploids (2n = 2x = 26) and 7 allotetraploids (2n = 4x = 52) [1, 3]. The modern cotton cultivars are allotetraploid with 26 chromosomes (*n* = 2x = 26), evolved through hybridization and domestication of A1-genome diploids *Gossypium herbaceum* (*n* = x = 13) with indigenous D5-genome diploids *G. raimondii*, a new world cotton species [4, 5] [6, 7].

Importantly, over 97% of the global cotton fiber production comes from the two main cultivated allotetraploid species, *G. hirsutum* L. and *G. barbadense* L [8]. In addition, the genus Gossypium contains over 50 species (45 diploid and 5 allotetraploid) including the most widely grown *G. hirsutum* and *G. barbadense*. This has led to extensive phenotypic diversity in cotton crops across many geographic regions worldwide [9]. However, the Egyptian cotton (*G. barbadense*) known by its extra-long and staple fiber Pima cotton characteristics is a famous cotton source for textile industry worldwide due to its unique chemical composition properties.

Traditional cotton breeding methods aim to improve cotton quality and yield by identifying high-performing parent lines with desirable agronomic traits for crosses. However, these classical techniques have limitations. More advanced molecular breeding utilizing genetic markers and genotyping could overcome these limitations and accelerate cotton improvement [5, 10, 11]. It is an important goal for cotton breeders to predict genetic similarities/dissimilarities and assess genetic diversity among cotton germplasm including genotypes, cultivated species, and wild relatives [12]. This allows accurate selection of potential lines to accelerate cotton improvement programs and obtain satisfactory yield and quality by maintaining sufficient genetic variability in cotton gene pools [13, 14].

Therefore, the lack of suitable genetic diversity in breeding germplasm is a major constraint slowing cotton breeders' progress in developing new cultivars [15–19]. Using molecular markers as an alternative tool to identify and select superior parents early in breeding programs and incorporate them into marker-assisted selection (MAS) could significantly enhance genetics and reduce time and costs required to develop novel cotton cultivars [12, 20–22].

Taking together, one of the main tools for cotton breeders to study the genetic diversity is using the molecular markers system that have already overcome the obstacles and disadvantages of morphological markers/characters that have a limit number and are affected by different plant growth stages as well as various environmental conditions [23–25]. In this regard, there are many types of DNA molecular markers that have been extensively used for various genetic analyses of cotton crop species such as RFLPs, RAPD, AFLPs, ISSRs, SSRs, and SNPs [26–29]. SSR markers, which are small motifs consisting of one to six tandem repeats, have been extensively and effectively utilized in genetic diversity, DNA fingerprinting, and QTL mapping studies for cotton crops. This is due to their distinctive DNA-based markers, which possess high specificity in amplifying genomic loci, a simple and easy operation system, a high degree of polymorphism, codominant nature, good reproducibility, and a wide distribution throughout the entire genome [26, 30, 31].

Given this context, the primary objective for cotton breeders is to create a publicly accessible database of molecular markers that can be used as a genetic diversity detection system. These markers, such as SSR markers, are closely associated with important agronomic and fiber quality traits. The aim is to expedite the process of selecting and breeding these traits to ensure sustainable cotton production [32, 33]. To date, genomic libraries contain over 1000 publicly available SSR designed primers from existing cotton DNA sequences generated by research groups worldwide [34, 35]. Many studies have used SSR markers to determine genetic diversity in diverse cotton germplasm. For example, Manonmani et al. [36] characterized genetic diversity of 12 Indian cotton genotypes using 55 SSR primer pairs. They found 40 pairs (25 polymorphic and 15 monomorphic) showed clear, scorable bands and an average of 1.8 alleles per locus [37].

The objective of this study was to explore and evaluate the molecular diversity and genetic polymorphisms among fourteen cotton genotypes using agronomic/morphological characters and genomic SSR markers. The goal was to identify suitable elite divergent genotypes that could be used as parents in future cotton breeding programs.

## Materials and Methods

### Experimental plant materials

A total of fourteen cotton (*Gossypium barbadense* L.) genotypes consisted of some Egyptian cotton genotypes and other foreign cotton cultivars were used for this investigation as experimental materials. The seed materials of these studied genotypes were obtained from Cotton Breeding and Genetics Department, Cotton Research Institute, Agricultural Research Center (ARC), Egypt. Details of these genotypes are presented in Table 1**.**

**Table 1:** Origin, and pedigree for the fourteen parental cotton genotypes utilize in this study

No.	Genotype	Pedigree	Origin
1.	Giza 86	G.75 × G.81	Egypt
2.	Giza 68	G.36 × G.56	
3.	Giza 96	G.84 × (G.70 × G.51B) × Pima62	
4.	Giza 94	10,229 × G.86	
5.	Giza 92	G.84 × (G.74 × G.68)	
6.	Giza 70	G.59A × G.51B	
7.	Giza 93	G.77 × PimaS6	
8.	Giza 45	G.28 × G.7	
9.	Karchenky Branches	Unknown	Russia
10.	Suvin	Sujata × Vincent	India
11.	Pima s_6_	5934–23–2 × 615,903–98–4–4	USA
12.	Pima high percentage	Unknown	
13.	C.B. 58	Unknown	
14.	Big Black Boll (B.B.B)	Unknown	Greek

### Experimental design and field assay

The field experiment for this study was conducted in the Agricultural Research Station in Sakha, which is part of the Egyptian governate of Kafr El-Shaikh. During the 2015 and 2016 growing seasons, this study used a randomized complete block design (RCBD) using triplicates.

#### Measurements of studied traits

Twelve morphological along with fiber traits were evaluated in the field as follows: 1. Position of first fruiting node (P.F.F.N); 2. Days to first flower (D.F.F); 3. Number of vegetative branches per plant (NO.V.B./P); 4. Number of fruiting branches per plant (NO.F.B./P); 5. Boll weight (B.W); 6.Seed cotton yield per plant (S.C.Y./P); 7. Lint yield per plant (L.Y./P); 8. Lint percentage (L.%) 9. Fiber length. (F.L); 10. Fiber fineness (F.F); 11. Fiber strength (F.S); 12. Uniformity ratio (UR). All morphological, agronomical and fiber traits/properties tests were measured according to known cotton measurement standards.

### DNA assay for diversity assessment

All molecular work related to this study was conducted in genetics and biotechnology laboratories, Faculty of Agriculture, Kafr El-Sheikh University and GEBRI, University of Sadat City, Egypt.

#### Genomic DNA samples collection, isolation, purification, and quantification

To assess the genetic diversity of these genotypes, the fresh leaves from each genotype were collected separately during the seedling growth stage. Then, pre-weighted leave tissue samples from 0.2 to 0.5 g were immediately frozen in liquid nitrogen and fully grounded to fine powder using a pestle and mortar. The subsequent steps of total genomic DNA extraction and purification were carried out using the CTAB method [38] with a few modifications. The isolated DNA samples were measured quantitatively using UV Mass spectrophotometer at a specific optical density (A_260_ and A_280_) as well as were qualitatively checked 1.5% agarose gel along with the standard DNA marker/ladder. The DNA samples were stored at –20 °C in a final concentration of 50 ng per microliter for further downstream steps.

#### PCR amplification, electrophoresis detection and polymorphism analysis protocols

The isolated genomic DNA samples from the 14 cotton genotypes were screened using 10 BNL series SSR primers/markers. These examined primers were obtained and designed based on available sequence information in the Cotton Marker Database (CMD) as summarized in Table 2. The DNA samples were amplified using polymerase chain reaction (PCR) in a 20 μl final reaction volume according to the method described by Saif et al. [39]. The PCR amplification reactions were conducted using 20 ng of DNA in a 25-μL reaction volume, comprising 0.3 μM of each primer, 200 μM of dNTPs, 5 μL (1X) of Taq polymerase buffer, 1.5 mM MgCl2, and 0.5 U Taq DNA polymerase. For SSR reactions, a Touchdown PCR program was employed. The primary program involved 9 cycles at 94ºC for 1 min, 54ºC for 1 min (with a 1ºC decrease in every cycle), and 72ºC for 1 min. Subsequently, 28 cycles were executed at 94ºC for 1 min, 45ºC for 1 min, and 72ºC for 1 min. The initial cycles were preceded by a denaturation step at 94ºC for 5 min and followed by an extension step at 72ºC for 5 min. Then, the PCR products (amplicons) were stored at 4 °C for the next step of gel electrophoresis. The amplified PCR products were separated by gel electrophoresis on 3% agarose gel [39]. The gel photos were visualized and taken under UV light using Gel Documentation System, and the bands were scored as 1 while the absence of a band was recorded as 0, and the [0,1] binary data matrix was constructed.

**Table 2:** Detailed summary of SSR primers involved in the molecular analysis of present study

Primer code	Forward sequence (5′-3′)	Reverse sequence (5′-3′)
BNL2823	ATATTCATGCCTCTGCAGCC	GTTTTTAGTTTTTGGACTTAGAGGC
BNL2827	ATCGCGGGCATTAATGAATA	AATACATCCGCTCATTTCGC
BNL1044	TGCTCTTTTTTGGGGGACTA	ATTGGCTTTGGTTGGTTGAG
BNL1440B	CCGAAATATACTTGTCATCTAAACG	CCCCCGGACTAATTTTTCAA
BNL3408A	ATCCAAACCATTGCACCACT	GTGTACGTTGAGAAGTCATCTGC
BNL3408B	ATCCAAACCATTGCACCAGC	GTGTACGTTGAGAAGTCATCTAT
BNL2634A	AACAACATTGAAAGTCGGGG	CCCAGCTGCTTATTGGTTTC
BNL193	TGTGAGCCATTGCTGTTAGC	TAAGTGCTGGCATTGTGAGC
BNL1047	GCTTGTCATCTCCATTGCTG	TAGCCCGGTTCATGTTCTTC
BNL1061	GCTTGTCATCTCCATTGCTG	TAGCCCGGTTCATGTTCTTC

### Data and genetic diversity analysis

The observed field data was analyzed to estimate the mean performance differences between studied cotton genotypes based on 12 agro-morphological and fiber collected traits using SPSS 21.0 software (https://www.ibm.com/products/spss-statistics). While the genotypic data based on SSR markers screening assay was analyzed as follows; For each cotton genotype, the amplified gel bands of each target SSR primer representing different alleles were scored, and the allelic bands reflecting the allelic variation were compared with 100 bp DNA ladder. Quantity one software (Gel Doc, Bio-Rad Laboratory, Inc.) was used for capturing gel images and the length of generated DNA fragments were estimated. Then, its data was converted into [0,1] binary matrix subjected to multivariate analysis. The polymorphism information content (PIC) analysis was estimated for all cotton genotypes based on their SSR markers allelic frequency according to the described method of Anderson et al. [40] which showed various PIC values [41] indicating different informative potential of each used SSR marker (High: more than 0.5; Moderate: between 0.5 and 0.25; Slightly below: 0.25.). The cluster dendrogram was constructed using UPGMA method based on the pairwise genetic distances [42] between the cotton genotypes using Numerical Taxonomy System, NTSYS-PC and NTSYS Pc 2.1 software [43]. Finally, the similarity matrices based on Jaccard similarity coefficients [44] were estimated by NTSYS-PC and NTSYS Pc 2.1 software.

## Results

### Agronomic and morphological characters

Table 3 displays the mean values of agro-morphological traits derived from the genotypes that were examined in the field. In general, the initial fruiting node location character showed the highest mean values from the Egyptian cotton genotypes; G.96 (8.33), G.68 (8.33), and G.93 (8.17), in that order. Conversely, the C.B. 58 genotype had the lowest mean value (5.42), while the Karchenky Branches and Suvin genotypes came in at 5.92 and 6.08, respectively. But in the genotypes that were considered, this feature varied between 5.42 and 8.33. While G.45 (25.58) had the best mean value for fruiting branches per plant, G.68 (22.17) and Suvin (20.08) were next ideal. On the other hand, genotypes G.93 (17.58), G.86 (17.75), and G.94 (17.92) yielded the lowest mean values.

**Table 3:** The mean performances of fourteen genotypes for earliness, growth habit, yield, and fiber quality traits for two years

Genotype	P.F.F.N	No.F.B.P	NO.V.B.P	D.F.F	B.W	S.C.Y.P	L.Y.P	L.%	F.L	F.S	F.F	U.R
**G.86**	7.92	17.75	3.58	74.05	3.05	108.70	42.61	0.3919	33.97	10.37	4.10	87.27
**G.68**	8.33	22.17	4.00	72.11	3.13	100.01	34.85	0.3489	35.93	10.67	3.63	86.70
**Pima s6**	8.08	17.92	3.83	70.71	3.06	96.08	34.78	0.3618	35.50	10.57	4.07	85.67
**Suven**	6.08	20.08	2.50	68.52	2.99	78.91	29.54	0.3744	34.50	10.37	4.03	84.23
**G.96**	8.33	18.08	3.17	70.16	3.31	97.71	38.28	0.3907	36.00	11.07	4.10	87.37
**G.94**	7.17	17.92	2.25	68.58	3.31	111.59	45.14	0.4047	34.53	11.20	3.90	87.57
**C.B. 58**	5.42	19.00	2.08	67.13	2.82	90.16	34.25	0.3800	34.63	10.50	4.10	84.67
**P.H.P**	6.58	18.33	2.08	66.59	2.85	80.79	32.01	0.3955	34.03	11.00	3.50	85.87
**G.92**	7.67	18.75	4.08	69.40	3.03	81.39	27.82	0.3433	33.83	11.50	4.00	86.87
**K.B**	5.92	18.67	2.33	67.79	3.05	68.75	24.67	0.3604	35.43	10.40	4.23	84.47
**G.70**	8.00	19.50	3.75	74.79	2.57	106.64	38.04	0.3567	36.80	11.40	3.90	88.10
**G.93**	8.17	17.58	3.33	70.18	2.84	102.40	35.07	0.3425	37.07	11.43	3.33	87.27
**G.45**	8.17	25.58	5.00	70.04	2.54	92.41	31.51	0.3412	36.83	11.17	3.27	86.97
**B.B.B**	6.58	19.25	2.42	67.00	3.15	101.14	40.81	0.4041	34.07	11.17	4.13	85.53
**L.S.D **_0.05_^*^	0.59	1.83	0.70	2.01	0.19	14.81	5.86	2.12	0.82	0.28	0.25	0.98
**L.S.D **_0.01_^**^	0.78	2.43	0.92	2.66	0.25	19.60	7.76	2.81	1.09	0.38	0.338	1.30

*P.F.F.;/* First Fruiting Node, *No. FB/P* Number of fruiting branches per plant, *No.V.B.P. *Number of vegititive branches per plant, *D.F.F* Days to first flower, *B.W.* Boll weight, *LCY.p.* Lint cotton yield per plant, *SCY.p* Seed cotton yieldper plant, *L%* Lint percentage, *F.L.* Fiber length, *FF* Fiber fineness, *FS* Fiber strength, *UR%* Uniformity ratio

^*^LSD0.05 = least significant differences of means (*p* < 0.05), **LSD0.01 = least significant differences of means (*p* < 0.01)

The highest mean values for the number of vegetative branches per plant were obtained from genotypes G.68 (5 branches), G.92 (4 branches), and G.45 (5 branches), as shown in Table 3. However, genotypes C.B. 58 (2.08), Pima high percentage (2.08), and G.94 (2.25), in that order, produced the lowest mean values. Curiously, the Egyptian genotypes G.70 (74.79), G.86 (74.05), and G.68 (72.11), in that order, had the highest mean values for the days to first flower trait. In contrast, the genotype Pima high percentage (66.59), B.B.B. (67), and C.B. 58 (67.13), in that order, had the lowest mean values.

#### Yield and its component characters

The mean values of yield and its associated parameters for all cotton genotypes are detailed in Table 3. In terms of boll weight (B.W), the data indicates that the genotypes G.96, G.94, and B.B.B exhibited the highest mean values at 3.31, 3.31, and 3.15, respectively, while the lowest mean values were observed in G.45, G.70, and C.B.58 at 2.54, 2.57, and 2.82, respectively. Analysis of seed cotton yield per plant (S.C.Y./P) in Table 3 reveals that the top-performing international cotton genotypes were Karchenky Branches, Suvin, and Pima high percentage, with mean values of 68.75, 78.91, and 80.79, respectively. Considering the lint yield per plant (L.Y./P), the data in Table 3 demonstrates that the genotypes G.94, G.86, and B.B.B exhibited the highest mean values at 45.14, 42.61, and 40.81, respectively. Conversely, the cotton genotypes Karchenky Branches, G.92, and Suvin recorded the lowest mean values at 24.67, 27.82, and 29.54, respectively. In terms of lint percentage (L.%), the results indicated that the genotypes G.94, B.B.B, and Pima high percentage had the highest mean values at 40.47, 40.41, and 39.55, respectively. On the other hand, the Egyptian cotton genotypes G.45, G.93, and G.92 exhibited the lowest mean values at 34.12, 34.25, and 34.33, respectively.

#### Fiber quality properties traits

In the case of cotton quality characteristics and properties, it was stated that the highest mean values for fiber length (F.L) trait were acquired from the Egyptian cotton genotypes, G.93, G.45 and G.70 (37.07, 36.83 and 36.8) respectively. Conversely, the lowest mean values were found from genotypes G.92, G.86 and P.H.P (33.83, 33.97 and 34.03), respectively. On the other side, the results in Table 3 exhibited that the highest mean values for the traits of fiber strength (F.S) were (11.5, 11.43 and 11.4) on the Egyptian genotypes G.92, G.93 and G.70, respectively whereas the lowest mean values were obtained from genotypes Suvin, G.86 and Karchenky Branches (10.37, 10.37 and 10.4), respectively.

In addition, the fiber fineness (F.F) character showed higher mean values from the cotton genotypes; Karchenky Branches, B.B.B and G.86 (4.23, 4.13, 4.1), respectively. While the lowest mean values were obtained from genotypes G.45, G.93 and P.H.P (3.27, 3.33 and 3.5), respectively. Finally, almost all the tested genotypes showed high ratio of uniformity but the highest mean values for uniformity ratio (U.R) trait were obtained from The Egyptian genotypes; G.70, G.94 and G.96 (88.1, 87.57 and 87.37) respectively. While the lowest mean values were attained from the Indian genotype (Suvin), the Russian genotype (Karchenky Branches), and one of American cottons (C.B. 58). Taking together, most of the Egyptian cotton genotypes showed high agronomic performance compared to the international genotypes.

### Molecular diversity revealed by SSR markers.

**Fig. 1:**
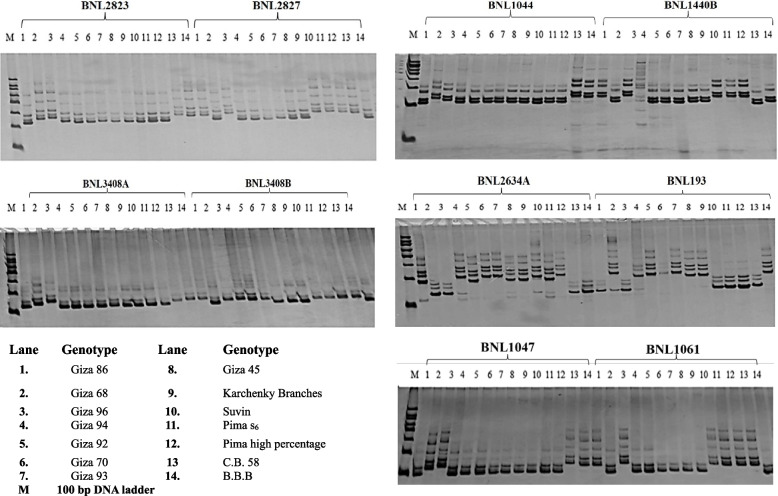
DNA fingerprints showed the polymorphism of fourteen cotton genotypes with ten SSR primers

Ten SSR markers, revealing a notably high level of polymorphism (polymorphic DNA), as outlined in Table 4 and Fig. 1. Across all tested cotton genotypes, the results indicated that the primer pairs designed for SSR analysis generated a total of 212 bands, with 175 of them being polymorphic. This accounted for 82.54% of the total bands, with an average of 17.5 polymorphic bands per marker. The number of bands varied between 5 and 8 for the primer pairs BNL2827 and BNL2823. The polymorphic bands percentage ranged from 72.4% for the primer pair BNL1440B to 100% for the primer pair BNL193. The Polymorphic Information Content (PIC) values for the SSR primer pairs ranged from 0.76 for BNL193 to 0.86 for BNL2827, with the latter recording the highest PIC value among the ten SSR markers. In summary, all examined SSR markers were deemed informative, collectively revealing an average PIC of 0.815.

**Table 4:** The diversity analysis results generated by ten simple sequence repeats (SSR) markers used in the study

No.	Marker name	Chr. No.	No. of allele	Polymorphic bands	PIC
1	BNL2823	6	8	16	0.84
2	BNL2827	1	8	12	0.86
3	BNL1044	4	5	18	0.81
4	BNL1440B	25	4	14	0.79
5	BNL3408A	3	3	12	0.80
6	BNL3408B	3	2	18	0.78
7	BNL2634A	7	6	19	0.83
8	BNL193	18	3	24	0.76
9	BNL1047	25	6	22	0.85
10	BNL1061	25	5	20	0.83
Total			**50**	**175**	**8.15**
Average			**5**	**17.5**	**0.82**

#### Similarity coefficient assessment

The genetic similarity co-efficient matrix of cotton genotypes used in this study (Table 5) showed that the similarity index (SI) values ranged from 0.5824 to 0.9066 with an average of 0.7473 as well as a high dissimilarity coefficient of 0.5824 and 0.6044 for the Egyptian genotype Giza 68 with genotypes Pima s_6_ and Pima high percentage, respectively. On the other hand, the highest similarity coefficient was recorded for the genotypes Giza 70 and Giza 93 (0.9066) and the genotypes Pima s_6_ and Pima high percentage respectively (0.9011). In addition to that, the Indian genotype (Suvin) recorded the higher genetic similarity index with the American genotypes; Pima high percentage (0.8901 followed by Pima s_6_ (0.8681) cotton respectively.

**Table 5:** Genetic similarity co-efficient matrix of 14 cotton genotypes based on genomic SSR molecular analysis

Genotype	Giza 86	Giza 68	Giza 96	Giza 94	Giza 92	Giza 70	Giza 93	Giza 45	K.P	Suvin	Pima s_6_	P.H.P	C.B. 58	B.B.B
**Giza 86**	**1.0000**													
**Giza 68**	0.6538	**1.0000**												
**Giza 96**	0.7747	0.7143	**1.0000**											
**Giza 94**	0.6868	0.6703	0.6923	**1.0000**										
**Giza 92**	0.6978	0.7363	0.6813	0.8242	**1.0000**									
**Giza 70**	0.6923	0.7198	0.7308	0.8077	0.8846	**1.0000**								
**Giza 93**	0.6868	0.7582	0.6703	0.7912	0.8462	0.9066	**1.0000**							
**Giza 45**	0.6648	0.6813	0.6703	0.7912	0.8242	0.8407	0.8571	**1.0000**						
**K.P**	0.6593	0.6758	0.6758	0.7857	0.8077	0.8462	0.8297	0.8956	**1.0000**					
**Suvin**	0.7527	0.6374	0.7692	0.7473	0.7253	0.7967	0.7582	0.7253	0.7198	**1.0000**				
**Pima s**_**6**_	0.7198	0.5824	0.7473	0.7802	0.7473	0.7857	0.7363	0.7692	0.8077	0.8681	**1.0000**			
**P.H.P**	0.7418	0.6044	0.7582	0.7802	0.7473	0.7967	0.7692	0.7692	0.7637	0.8901	0.9011	**1.0000**		
**C.B. 58**	0.6319	0.7363	0.6813	0.6703	0.7143	0.7308	0.7363	0.7033	0.6923	0.7088	0.7418	0.7418	**1.0000**	
**B.B.B**	0.6319	0.7363	0.6813	0.6703	0.7143	0.7308	0.7363	0.7033	0.7198	0.6154	0.6374	0.6593	0.7747	**1.0000**
	**Maximum: 0.9066**					**Average:0.7473**						**Minimum:0.5824**		

#### Cluster analysis

**Fig. 2:**
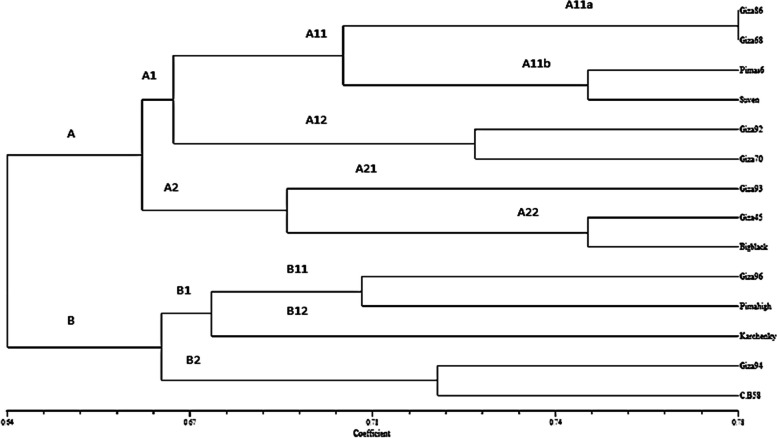
Cluster analysis dendogram constructed from the studied cotton genotypes through ten SSR primers

For this study, the fourteen cotton genotypes were scored based on the presence and absence of amplified band for each SSR marker and its specific alleles. Thus, genetic distance analysis showed that for each genotype combination, the genetic distance ranged from 0.64 to 0.78 and according to the cluster analysis of combined SSR data, all 14 genotypes used in this study were separated into two major clusters (Fig. 2). The constructed dendrogram has grouped the used genotypes into two distinguished clusters namely, A and B. According to the phylogenetic tree, it shown in Fig. (2) that the genetic similarity of 0.66 was the start separation point for main cluster to two sub clusters A1 and A2, the first sub cluster consisted of A11 and A12 at genetic similarity of 0.70, the A12 sub cluster included Giza 92 and Giza 70 at genetic similarity of 0.73. The A12 sub cluster separated to A11a and A11b, the A11a included G.68 and G.86 at genetic similarity 0.78 while the A11b included Pima s_6_ and Suvin at genetic similarity 0.75. The A2 sub cluster separated to A21 and A22, the A21 included individual cultivars G.93, while A22 included G.45 and B.B.B genotypes at genetic similarity 0.75. On the other side, the second main cluster was separated into two sub main clusters namely, B1 and B2 at the genetic similarity of 0.66. Then, the first sub cluster B1 was subsequently divided into another sub clusters of B11 and B12 at genetic similarity 0.71 while, The B2 included two genotype G.94 and C. B. 58 at genetic similarity 0.72. The B11 included G.96 and Pima high percentage at genetic similarity 0.71, while B12 included only one cultivar Karchenk Branches.

## Discussions

One of the essential goals for cotton breeders, is to develop modern varieties with promissing characteristiques in terms of fiber quality as well as agronomical economic traits to increase the farmers profitability under its current cultivation system. Unfortantely, the achievement of this goal is hindered by the poor and tapered of genetic base of modern crop varieties due to the continued extensive selection process during its progress course that eventually has leaded to a lack genetic variability amongest the core cotton genotypes [17, 37, 45]. Therefore, the current investigation was aimed to estimate the genetic diversity/variability among different Egyptian and international cotton genotypes using important agro-morphological traits and DNA based SSR markers.

The results of our investigation suggest that the Egyptian cotton genotypes demonstrated the most elevated mean performance values across all assessment criteria for growth performance. On the other hand, the genotypes G.96, G.94, B.B.B, and G.70 demonstrated the highest average values for both yield and its constituent components. The cotton genotypes G.96, G.92, Karchenky, G.94, G.93, B.B.B, and G.70 had the greatest average values for fiber qualities when compared to the other cotton genotypes. Likewise, a multitude of previous studies have demonstrated comparable patterns in the assessed agro-morphological parameters [46, 47].

The results obtained from the analysis of fourteen cotton genotypes using ten SSR/microsatellite molecular markers are of significance. It is crucial to note that the effectiveness of various DNA-based markers in assessing genetic variation in crops can vary based on genetic principles and the rationale behind using each molecular marker [48]. In our genetic diversity analysis, as depicted in Table 4, the total and average number of polymorphic bands for the studied SSR markers were found to be higher compared to the findings of Kurt et al. [30] In another study, they analyzed twenty-nine genotypes, including interspecific hybrid cotton, using twelve genomic SSR markers. They observed a different number of amplified alleles ranging from 2 to 4 for each locus, with an average of 2.53 alleles per locus. [12, 41, 49–51].

Moreover, the investigation carried out by Dongre et al. [52] found that out twenty-five25 SSR markers tested in their study, 17 markers were able to produce 56 polymorphic bands in addition to four SSR markers showed a monomorphic pattern while the remaining markers were non-scorable and non-reproducible bands. Taking together, the similar findings by using genomic SSR markers have been reported by various researchers such as [26, 31, 53–55]. In our investigation, the Polymorphic Information Content (PIC) values for all analyzed SSR markers varied, ranging from 0.76 for the primer pair BNL193 to 0.86 for the primer pair BNL2827, with an average PIC of 0.82. Additionally, it is crucial to emphasize that the discerned genetic diversity in the examined germplasm materials is not solely indicated by the varying number of amplified alleles for each marker. It also correlates with other factors, such as the type of marker system utilized, the separation technique of PCR products, and the resolution power of the analysis [56].

On the other side, the genetic similarity co-efficient and phylogenetic analysis results were figured out the genetic relationships amongst the studied cotton genotypes. These results were based on the molecular profiling data of examined cotton genotypes and it might be help to design as well as to conduct a hybridization-based breeding programs with the wide clustered related genotypes [57]. For example, as per our results, the hybridization between the Egyptian cotton genotypes such as Giza 68 with genotypes Pima s6 or Pima high percentage is a suitable parental combination in next breeding schemes due to the high dissimilarity coefficient between these genotypes. With this respect, SSR markers are a highly preferable tool to characterizes different crop genotypes to describe their expansion regarding its genetic diversity as well as it is the suitable choice marker system to assess DNA-based fingerprinting for the major crop improvement schemes [58, 59]. In addition, the higher genetic variability in cotton genotypes was recorded through the implementation of SSR based markers system in cotton genetic diversity analysis and marker assisted selection studies [60]. On the other hand, according to Ditta et al. [41] It was asserted by the individual that a PIC value exceeding 0.5 for each SSR marker indicated the informative capacity of said marker. The findings of our investigation revealed that the polymorphism information content (PIC) results indicated a PIC value over 0.5 for all SSR markers that were evaluated. Thus, in summary, these SSR markers can serve as a valuable tool for cotton crop breeders to investigate the genetic diversity and expand the genetic resources of cotton. This will help identify appropriate parental lines and establish a strong foundation for future marker assisted selection (MAS) schemes aimed at enhancing new modern cotton genotypes.

## Conclusion

In conclusion, the superior performance of Egyptian cotton genotypes, particularly G.96, G.68, and G.93, in key agronomic traits underscores their potential for cultivation and breeding programs. The robust fiber quality traits exhibited by these genotypes further highlight their significance in contributing to high-quality cotton production. The molecular analysis, using SSR markers, not only revealed a substantial level of genetic polymorphism but also facilitated the identification of distinct genetic relationships among the studied genotypes. This comprehensive understanding of both phenotypic and genotypic characteristics provides valuable insights for cotton breeders and farmers, aiding in the selection and development of improved cotton varieties with enhanced agronomic performance and fiber quality.

## Data Availability

All data generated or analysed during this study are included in this published article.
